# Spatiotemporal patterns of soil myxomycetes in subtropical managed forests and their potential interactions with bacteria

**DOI:** 10.1128/aem.00479-25

**Published:** 2025-05-13

**Authors:** Wen-Long Song, Di Lin, Xia Chen, Qun Dai, Gu Rao, Ya-Jing Chen, Shuang-Lin Chen

**Affiliations:** 1 School of Life Sciences, Nanjing Normal University 224704, Nanjing, Jiangsu, China; 2 Dr. Sun Yat-Sen Mausoleum Administration, Nanjing, Jiangsu, China; 3 School of Life Sciences and Chemical Engineering, Jiangsu Second Normal University 66471, Nanjing, Jiangsu, China; Colorado School of Mines, Golden, Colorado, USA

**Keywords:** Amoebozoa, community assembly, forest types, metabarcoding, myxamoeba, seasons, 16S rDNA, 18S rDNA

## Abstract

Soil myxomycetes are crucial soil protists with important ecological functions. Yet, our understanding of their diversity patterns in managed forests and the interactions with their food is far behind other taxa. This study investigates the spatiotemporal patterns of soil myxomycetes in four northern subtropical managed forest types across seasons and aims to identify assembly processes, main predictors of myxomycete communities, and the potential interactions between myxomycetes and bacteria. Results showed that no significant difference in α diversity of myxomycete communities among forest types was observed, but a significant difference was observed in community structures. Significant differences were observed in α diversity and community structures of myxomycetes among seasons. Deterministic processes in each forest type and season dominated myxomycete community assemblies. Soil physicochemical properties and bacterial communities have a significant direct impact on the myxomycete community, while forest types, seasons, and enzyme activities have an indirect effect. There is a significant synergistic covariation between the soil myxomycete community and bacterial community. The genera of the phyla Acidobacteriota, Actinobacteriota, and Bacteroidota have a strong correlation with the richness of myxomycete genera. Overall, this study provides new insight into the diversity of soil myxomycetes and their influence by bacteria, crucial for myxomycetes ecology.

**IMPORTANCE:**

Soil myxomycetes are an important component of soil protists. Our study revealed for the first time the community structure of soil myxomycetes in managed forests of the northern subtropical regions and systematically investigated the seasonal variation patterns of soil myxomycetes. Meanwhile, we further investigated the potential interactions between soil myxomycetes and bacteria. This study will greatly enhance our understanding of the ecology of soil myxomycetes and their biological roles.

## INTRODUCTION

Myxomycetes, one of the largest taxonomic groups within Amoebozoa of Protist (1), constitute a critical component of soil protist diversity (2). These organisms fulfill essential ecological functions by decomposing organic matter, stimulating microbial activity, regulating microbial communities, and facilitating nutrient cycling (3). The life cycle of myxomycetes includes two trophic stages (myxamoeba and plasmodium) and a reproductive stage characterized by fruiting body formation. The class Myxomycetes comprises two subclasses: dark-spored clade (Columellomycetidae) and bright-spored clade (Lucisporomycetidae) (4), encompassing over 1,100 species (5). Current knowledge of myxomycete diversity and ecology predominantly derives from fruiting body surveys (6), leaving the cryptic myxamoeba stage severely understudied (7).

Recent advances in 18S rDNA high-throughput sequencing have enabled targeted investigations into soil dark-spored myxomycete communities during specific temporal windows (2, 7–12). Nevertheless, critical gaps persist in spatiotemporal dynamics of soil myxomycete community composition, assembly mechanisms, and environmental driving factors, which lag far behind analogous research on other microbial groups (7). Limited evidence suggests that a community assembly in myxomycetes is mainly governed by deterministic processes (7), though randomness, such as dispersal limitation, also exerts a measurable influence (8, 10).

Bacteria are an important food source for soil protists. As bacteriophagous protists (13, 14), myxomycetes occupy a keystone position in the soil food web by regulating bacterial populations. The potential predator-prey interactions between myxomycetes and bacteria may drive niche partitioning, as evidenced by correlations between myxomycete community structure and specific bacterial/fungal taxa (2). In addition, different myxomycete plasmodia exhibit different bacterial community compositions, suggesting that myxomycetes may positively recruit certain kinds of bacteria from the surrounding environment (15). However, the bidirectional ecological linkages between myxomycetes and bacteria remain poorly understood.

At present, research on the diversity of myxomycetes has focused overwhelmingly on pristine forest ecosystems (2, 7–10, 16) characterized by minimal anthropogenic disturbance and intact vegetation profiles. In contrast, managed forests, which are frequently subject to human intervention through activities like recreational use, selective logging, or proximity to urban areas, have received considerably less research attention (17), especially in myxomycetes (10, 18). Managed forests as a transitional zone between wilderness and human habitats have important ecological significance, and their ecological functions are increasingly receiving attention (19, 20). As key decomposers of forest ecosystems, understanding the community characteristics and spatiotemporal dynamic changes of myxomycetes in managed forests is significant for evaluating transitional zone ecosystems.

In this study, we aim to enhance our understanding of the diversity and ecology of soil dark-spored myxomycetes by addressing the following questions: (i) How do forest type and seasonality shape dark-spored myxomycete community structure and assembly in managed forest soil? (ii) What environmental factors predominantly govern these communities? (iii) Do covariation patterns exist between dark-spored myxomycetes and bacteria? To answer these questions, this study employed 18S rDNA and 16S rDNA high-throughput sequencing to investigate the distribution patterns and dynamic changes of soil dark-spored myxomycetes and bacteria in four typically managed forest types in northern subtropical regions across four seasons. By quantifying deterministic versus stochastic assembly processes and evaluating drivers spanning forest characteristics, soil properties, enzyme activity, seasonality, and bacterial communities, this study clarifies the ecological process structuring myxomycete communities and identifies putative myxomycete and bacteria interactions.

## MATERIALS AND METHODS

### Study area, sites, and sampling

**Fig 1:**
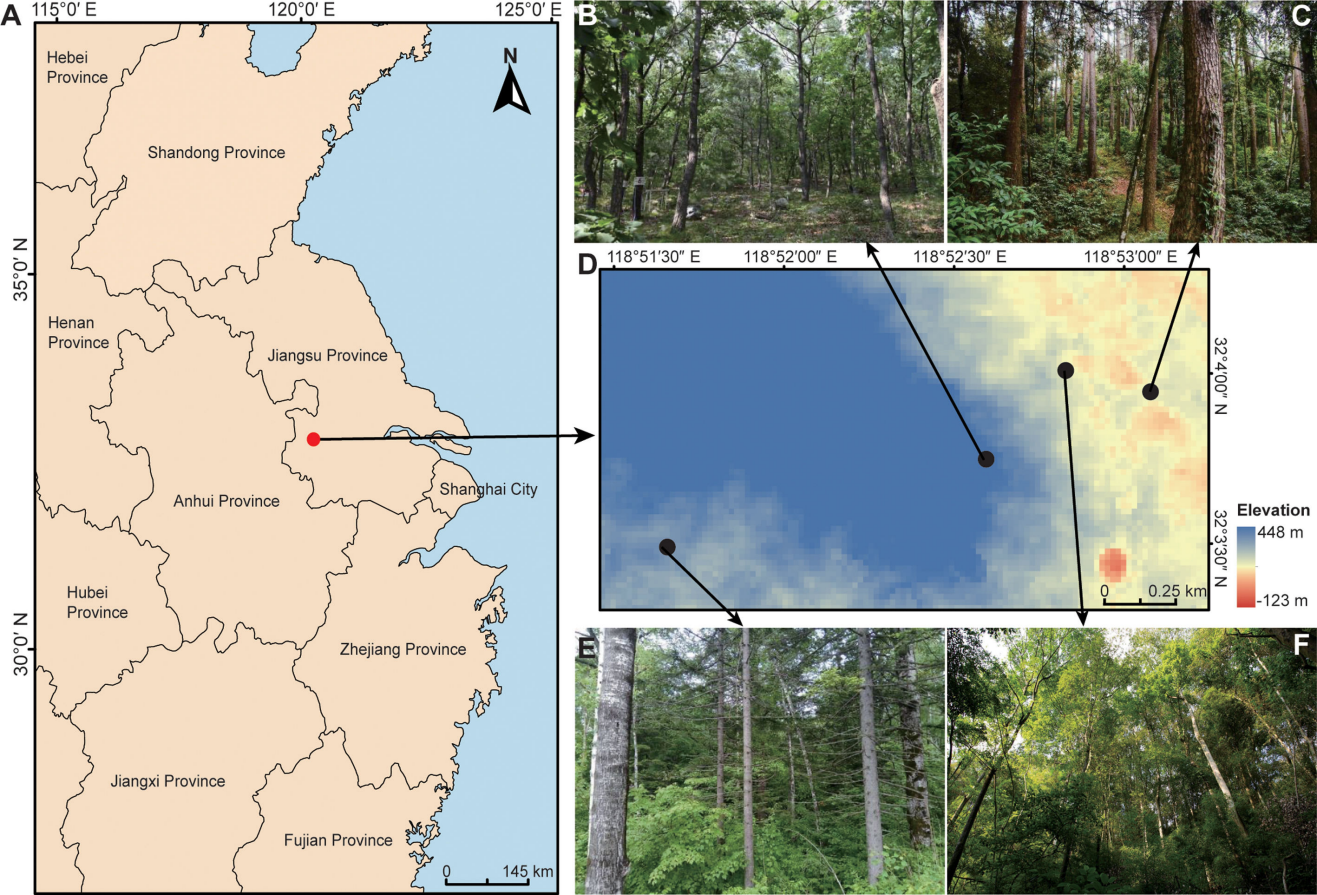
Geographical location of the research area and sampling points in this study. (**A**) Geographical location of Zijin Mountain National Forest Park in China. Map data sourced from National Platform for Common Geospatial Information Services (https://www.tianditu.gov.cn/). (**B**) Broad-leaved forest. (**C**) Coniferous forest. (**D**) Distribution of the four forest types selected in this study in Zijin Mountain National Forest Park. (**E**) Mixed coniferous and broad-leaved forest. (**F**) Mixed broad-leaved forest.

This study was conducted within planted forests of Zijin Mountain National Forest Park (32°01′–32°06′ N, 118°48′–118°53′ E, elevation 59–230m) in Nanjing, Jiangsu Province, China. The region has a subtropical monsoon climate with a mean annual temperature of 15.8℃ and a mean annual precipitation of 1,048mm (21). Four dominant forest types that co-occur in this area with varying plant species diversities and compositions were selected: a broad-leaved forest (BF) dominated by *Quercus variabilis* Blume, a coniferous forest (CF) dominated by *Pinus massoniana* Lamb., a mixed coniferous and broad-leaved forest (CBF) dominated by *Liquidambar formosana* Hance and *P. massoniana*, and a mixed broad-leaved forest (MBF) dominated by *Aphananthe aspera* (Thunb.) Planch. and *Celtis tetrandra* Roxb. These forests exemplify the regional features of afforestation and reforestation in southern China (21, 22). The geographical locations of the sample plots are shown in Fig. 1, and elevation and vegetation details are provided in Table 1.

**TABLE 1:** Information on the geographical location and vegetation of sampling sites

Site code	Broad-leaved forest (BF)	Mixed coniferous and broad-leaved forest (CBF)	Coniferous forest (CF)	Mixed broad-leaved forest (MBF)
Elevation (m)	62	59	100	165
Stand age (yrs)	60	50	70	70
Slope aspect	South	South	West	South
Dominant trees	*Quercus variabilis*	*Liquidambar formosana, Pinus massoniana*	*P. massoniana*	*Aphananthe aspera, Celtis tetrandra*
Understory Dominant plants	*Ardisia japonica*, *Lysimachia clethroides*	*Rubus hirsutus*, *Begonia fimbristipula*	*R. hirsutus*, *Arthraxon hispidus*	*Lonicera japonica*, *B. fimbristipula*

Five replicate 25 × 25m sample plots were established per forest type. Within each plot, five soil cores (0–10cm depth after removing the litter layer) were collected by the diagonal sampling method, sieved with a 2mm mesh, and then mixed as one sample (7). All soil samples were delivered to the laboratory with dry ice within 12h, where they were then divided into three parts: one was stored at −80℃ until DNA extraction, one was fresh soil sample for determining soil moisture content, and the other was used for measuring soil physicochemical factors and enzyme activity after natural air-drying. This study collected soil samples from four forest types in September 2022 (Autumn), December 2022 (Winter), March 2023 (Spring), and June 2023 (Summer). In total, 80 soil samples were collected (four forest types × four seasons × five plots).

### Determination of environmental factors and enzyme activity

Soil samples were air-dried and analyzed for soil water content (SWC), pH, total carbon (TC), and total nitrogen (TN) using standard methods (9). Available phosphorus (AP), available potassium (AK), soil electrical conductivity (EC), and soil organic matter (SOM) were measured following the methods previously described (23). Additionally, invertase (S-SC), cellulase (S-CL), acid proteinase (S-ACPT), and acid phosphatase (S-ACP) activities of soil were determined separately using the BC5240-50T/24S, BC0150-50T/24S, BC0860-50T/24S, and BC0145-100T/96S soil enzyme activity detection kit by Beijing Solarbio Science & Technology Co., Ltd. (Beijing, China).

### DNA extraction and sequencing

Soil genomic DNA was extracted from 1.5 g soil (fresh weight) of each soil sample using MagPure Soil DNA LQ Kit (Guangzhou Magen Biotechnology Co., Ltd., Guangdong Province, China) following the manufacturer's instructions. The DNA quality and concentration were evaluated using a NanoDrop NC2000 spectrophotometer (Thermo Fisher Scientific Inc., Waltham, MA, USA). The dark-spored myxomycetes 18S rDNA V2 region and bacterial 16S rDNA V3–V4 region were amplified with primers S31R/S3b (11) and 338F/798R (24). The polymerase chain reaction (PCR) products were purified, quantified with Qubit dsDNA Assay Kit (Thermo Fisher Scientific, Waltham, MA, USA), and pooled in equal amounts. All the amplicons were sequenced on the Illumina MiSeq PE300 platform (Illumina Inc., California, USA) at the Shanghai OE Biotech Co., Ltd. (Shanghai, China). The raw data generated in this study were deposited in the Sequence Read Archive under accession numbers PRJNA1241702 (Myxomycetes) and PRJNA1241720 (Bacteria).

### Sequence processing

Raw sequencing data were in FASTQ format. Paired-end reads were then preprocessed using Trimmomatic 0.35 (25) to detect and remove ambiguous bases (N). It also trimmed off low-quality sequences with an average quality score below 20 using a sliding window trimming approach. After trimming, paired-end reads were assembled using Flash 1.2.11 (26). Assembly parameters were set as follows: a minimum overlap of 10 bp, a maximum overlap of 200 bp, and a maximum mismatch rate of 20%. The split_libraries 1.8.0 (27) tool in QIIME was used to filter out sequences containing N bases, sequences with single base repetitions greater than eight, and sequences with lengths less than 200 bp from the spliced sequence. UCHIME 2.4.2 (28) was then employed to remove chimeric sequences. Operational taxonomic units (OTUs) were clustered using Vsearch 2.4.2 (29) based on sequence similarity, with a 98% cutoff for myxomycetes (2) and a 97% cutoff for bacteria. All low abundant OTUs (<10 sequences) were filtered out unless they achieved a 100% match to a reference sequence, as they may contain PCR or sequencing errors (7).

Taxonomic classification of the myxomycete OTUs was performed by aligning the sequences of known myxomycetes species in GenBank using the NCBI's BLAST tool. The myxomycete OTUs showing >98% similarity were assigned to species level, >95% similarity were assigned to genus level (30), >75% similarity were roughly assigned to family level (7), while those <60% were discarded to avoid overestimation of diversity metrics (7, 9). Taxonomic classification of the bacterial OTUs was conducted by querying the representative sequences against the SILVA database (31), using the RDP classifier Naive Bayesian classification algorithm (32) with a 99% similarity threshold.

### Statistical analysis

All analyses were performed in the R software environment (v 4.1.2) unless otherwise stated.

To address distinct hypotheses about forest and seasonality effects, we adopted two analytical groups: (1) forest type comparison: pooling all seasonal replicates (*n* = 20 per forest type) to assess overarching differences independent of temporal variation; and (2) seasonal comparison: pooling all forest type replicates (*n* = 20 per season) to assess overarching differences independent of forest type.

To compare soil physicochemical properties and enzyme activities across forest types and seasons, we implemented one-way analysis of variance (ANOVA) with the least significant difference (LSD) *post hoc* in GraphPad Prism 10. Myxomycete OTU distribution patterns among forest types and seasons were visualized using the Evenn (33). Rarefaction curves based on OTU richness per forest type and season were computed and visualized using incidence frequency data through the “iNEXT” R package (34). To eliminate the effects of different sequencing depths on statistical analyses, we rarified the sequence numbers of each sample to the sample with the lowest number of reads. The observed number of OTUs (OTU richness), Shannon index, and Pielou’s evenness index were used to represent the taxonomic richness, diversity, and evenness of myxomycete communities, with subsequent one-way ANOVA and LSD multiple comparisons conducted in GraphPad Prism 10.

To quantify the β diversity of myxomycete community composition between soil samples of different forest types or seasons, we calculated the Morisita-Horn similarity index (MH) (35) of myxomycete community between forest types and seasons using “dplyr” and “purrr” R package and visualized them by heatmap using GraphPad Prism 10. The calculation was $C_{MH}=\frac{2\sum_{i=1}^{S}\left(a_{ij}a_{ik}\right)}{\left(\frac{\sum_{i=1}^{S}a_{ij}^{2}}{A_{J}^{2}}+\frac{\sum_{i=1}^{S}a_{ik}^{2}}{A_{k}^{2}}\right)\times A_{j}A_{k}}$, where *a_ij_* and *a_ik_* are abundance of species *i* in communities *j* and *k*, *A_j_* and *A_k_* are total abundance of all species in communities *j* and *k*, and *S* is total number of species. This method takes into account the abundance of species. To further determine the ecological patterns of forest type and season assemblages, we conducted two principal coordinate analyses (PCoAs) based on Bray-Curtis similarities distances, which were calculated with log-transformed absolute abundance data using the “vegan” R package (36). The statistical significance of the PCoA ordination was further tested by permutational multivariate analysis of variance (PERMANOVA), which was performed with 999 permutations, and *post hoc* pairwise PERMANOVA using the “pairwiseAdonis” R package (37).

Sloan's neutral community model (38) and normalized stochasticity ratio (39) were employed to quantify the relative contribution of stochastic versus deterministic processes to myxomycete community assembly. In Sloan's neutral community model, the coefficient of determination (R^2^) reflects the model fit, while lower R^2^ values indicate stronger deterministic influence and weaker randomness. The parameter Nm [meta-community (N) × migration rate (m)] inversely correlates with dispersal limitations, with higher values suggesting reduced barriers to microbial exchange. For normalized stochasticity ratio interpretation, we adopted the modified stochasticity ratio, which calculated and corrected the normalized stochasticity ratio, where the modified stochasticity ratio >0.5 signifies deterministic dominance, whereas the modified stochasticity ratio <0.5 indicates stochastic-driven community assembly.

To assess environmental drivers of soil dark-spored myxomycete communities, we performed Mantel tests using the “linkET” R package, correlating myxomycete OTU (representing myxomycete community) with soil physicochemical properties, enzyme activities, and other influencing factors via Spearman's rank coefficients. Structural equation modeling (SEM) was subsequently applied to disentangle the direct and indirect effects of forest type, soil physicochemical factor, enzyme activity, seasonal variation, and bacterial communities on myxomycete communities (40).

To further explore the potential interplay between soil myxomycetes and bacteria, we performed linear regression analysis using GraphPad Prism 10 to assess the relationships between myxomycete OTU richness and bacterial OTU richness, as well as cross-correlations between their respective Pielou's evenness index and Shannon indices. To explore ecological covariation patterns, we applied co-inertia analysis utilizing the “ade4” package, projecting myxomycetes and bacteria variables into a shared low-dimensional space to visualize their covariations effectively (41). Subsequently, to examine potential interactions between myxomycetes and bacteria in greater detail, we first computed correlation coefficients between all annotated myxomycetes genera and the top 30 annotated bacterial genera (42) and evaluated the explanatory power of the top 30 annotated bacterial genera in predicting the relative abundance of myxomycetes at the genus level using a random forest model (43). Then, at the OTU level, we first filter out myxomycete and bacterial OTUs present in fewer than 20% of all soil samples before conducting a correlation analysis between myxomycete OTUs and bacterial OTUs. We retained correlations with an absolute value greater than 0.5 (|Spearman's r| > 0.5) (44) and statistical significance (*P* < 0.05) after the Benjamini-Hochberg correction. The results were visualized using Gephi 0.10.1 (45), by constructing an undirected weighted network with edge weights representing correlation magnitudes, applying the Fruchterman Reingold layout algorithm for optimal node positioning, and color-coding nodes by Myxomycetes and Bacteria while sizing nodes proportionally to interaction degrees.

## RESULTS

### Overview of soil physicochemical factors and enzyme activity

**Fig 2:**
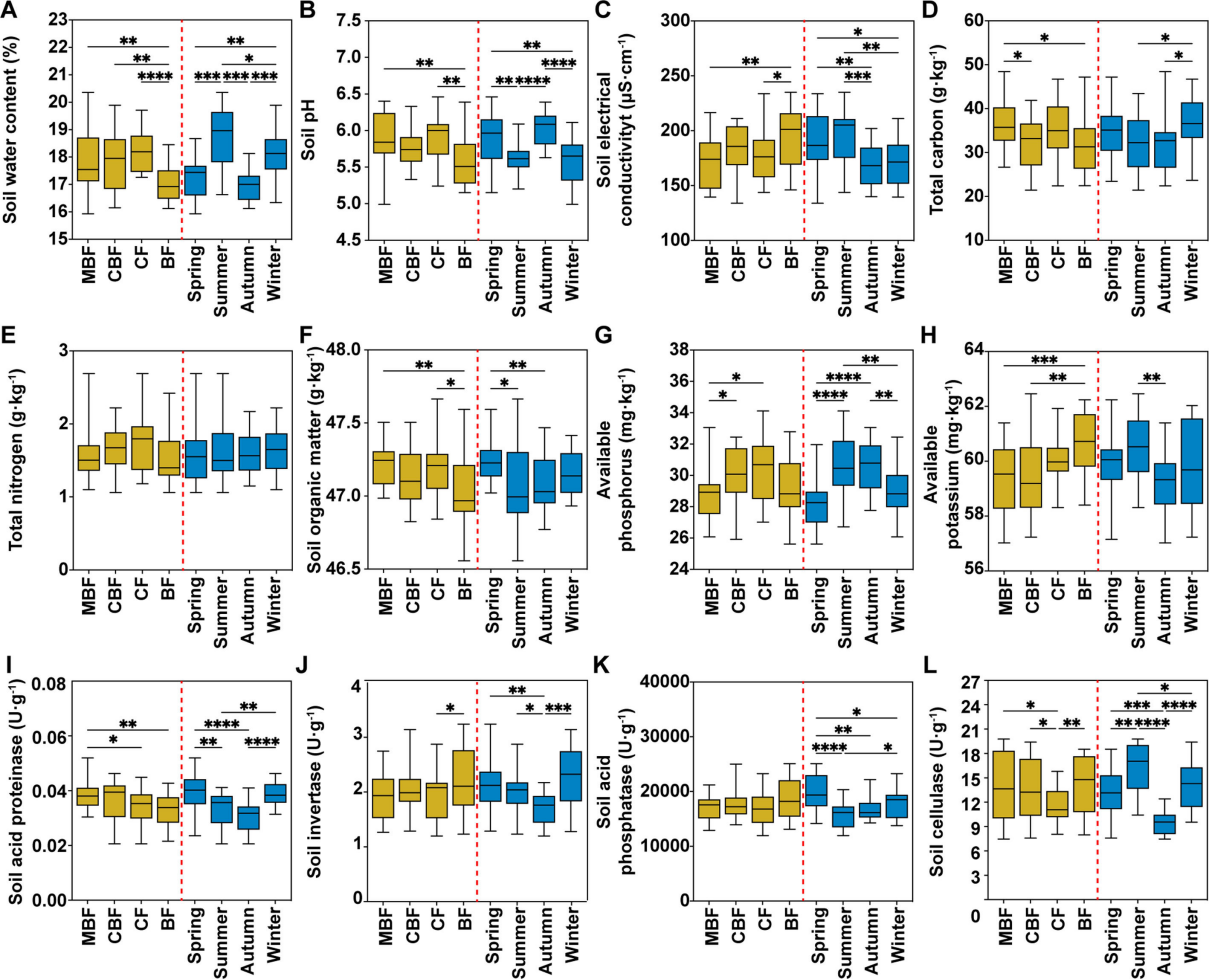
Physicochemical properties and enzyme activities of soil samples from four forest types and four seasons in the Zijin Mountain National Forest Park. (**A**) Soil water content. (**B**) Soil pH. (**C**) Soil electrical conductivity. (**D**) Soil total carbon. (**E**) Soil total nitrogen. (**F**) Soil organic matter. (**G**) Soil available phosphorus. (**H**) Soil available potassium. (**I**) Soil acid proteinase. (**J**) Soil invertase. (**K**) Soil acid phosphatase. (**L**) Soil cellulase. Each boxplot includes 20 data (*n* = 20), where the box spans the interquartile range (25th to 75th percentile), the central line denotes the median, and the whiskers extend to the minimum and maximum values. A one-way ANOVA with the least significant difference (LSD) was performed to compare the significant differences. The asterisks showed the *P* value significance level: *P < 0.05, **P < 0.01, ***P < 0.001, and ****P < 0.0001. Abbreviations used are as follows: BF for broad-leaved forest, CF for coniferous forest, CBF for mixed coniferous and broad-leaved forest, and MBF for mixed broad-leaved forest.

All soils were acidic pH (5.58–5.88) across forest types (Fig. 2B). The SWC was the highest (18.30 ± 0.80%) in CF (Fig. 2A). The EC in BF (193.2 ± 27.82 μS·cm^−1^) significantly exceeded other forests (*P* < 0.05; Fig. 2C). The TC and SOM in MBF (36.50 ± 5.68 g·kg^−1^ and 47.21 ± 0.15 g·kg^−1^, respectively) were substantially higher (*P* < 0.05) compared to other forest types (Fig. 2D and E), while TN showed no significant inter-forest variation (Fig. 2F). The AP differed significantly between MBF and CF (*P* < 0.05; Fig. 2G), whereas AK was the highest in BF (60.71 ± 1.12 mg·kg^−1^, *P* < 0.05; Fig. 2H). Although no forest type differences emerged in soil enzyme activities (Fig. 2I through L), LSD test showed that the S-ACPT in MBF (0.038 ± 0.005 U·g^−1^) was significantly higher than that in CF and BF (*P* < 0.05), and S-CL in CF (11.46 ± 2.04 U·g^−1^) was significantly lower than MBF and BF (*P* < 0.05).

For the seasons, all soil physicochemical properties except TN varied significantly by season (*P* < 0.05; Fig. 2). The SWC, EC, and AK showed peaks in summer (18.77 ± 1.06%, 195.5 ± 25.16 μS·cm^−1^, and 60.47 ± 1.22 mg·kg^−1^, respectively). The soil pH and AK were the highest in autumn (6.03 ± 0.22 and 30.59 ± 1.53 mg·kg^−1^, respectively). The TC in winter was the highest (36.60 ± 6.11 g·kg^−1^), and the SOM in spring was the highest (47.25 ± 0.15 g·kg^−1^). Soil enzyme activities peaked seasonally (Fig. 2I through L): spring dominated the S-ACPT (0.039 ± 0.006 U·g^−1^) and S-ACP (18,955.11 ± 5,647.69 U·g^−1^, *P* < 0.05), winter surpassed summer and autumn in S-SC (2.27 ± 0.57 U·g^−1^, *P* < 0.05), and summer showed maximal S-CL (16.28 ± 2.95 U·g^−1^, *P* < 0.05).

### Composition and α diversity of myxomycete community

**Fig 3:**
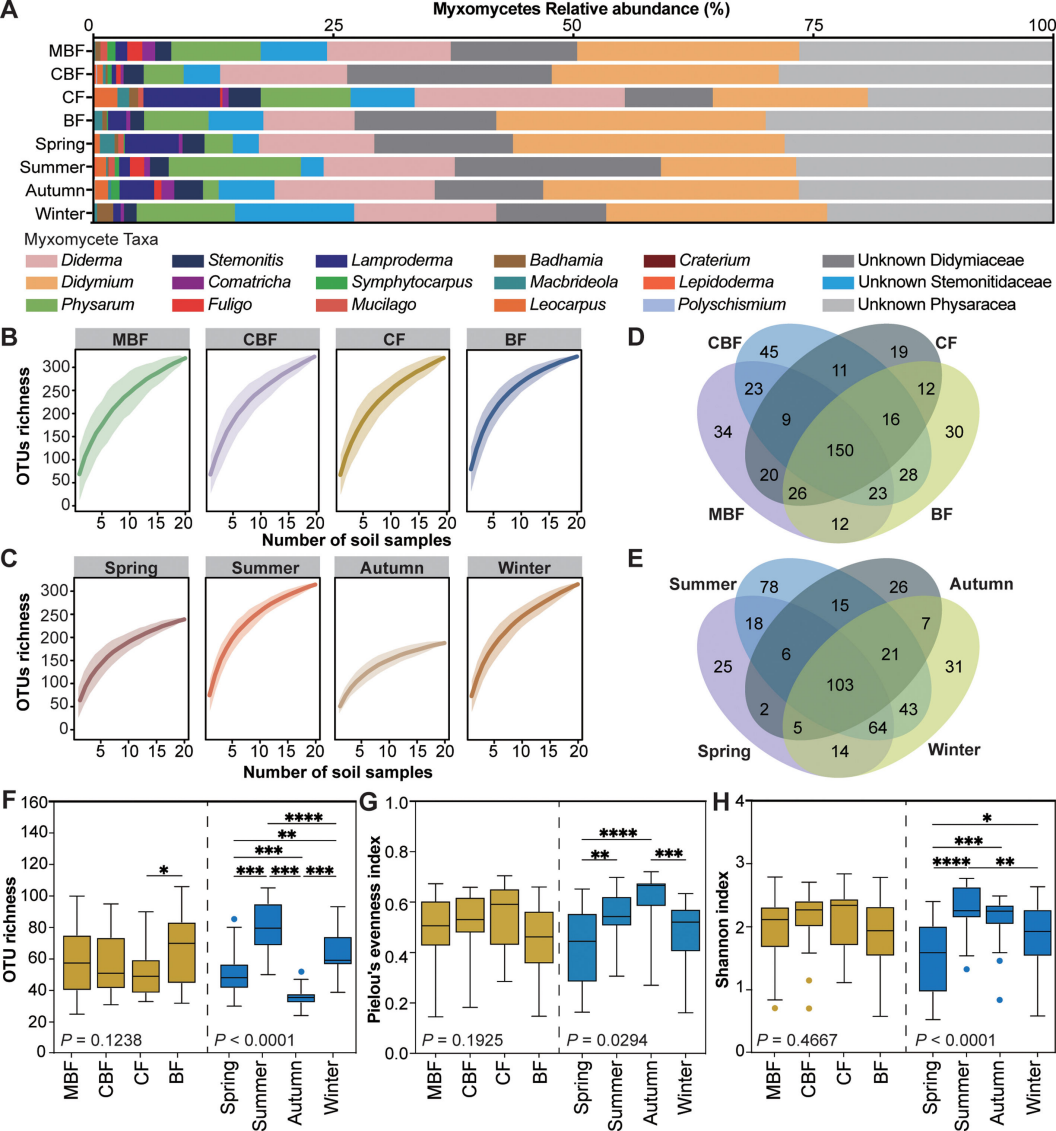
α Diversity of soil myxomycete communities. (**A**) Genus-level relative abundance across forest types and seasons. (**B and C**) Species accumulation curves based on incidence frequency of OTUs (solid: observed; dotted: extrapolated) across forest types (**B**) and seasons (**C**) with 100 bootstrap confidence intervals (CI) (shaded). (**D and E**) Shared OTUs among four forest types (**D**) and seasons (**E**). (F–H) Diversity indices across forest types and seasons: OTU richness (**F**), Pielou's evenness index (**G**), and Shannon index (**H**). Each boxplot includes 20 data (*n* = 20). A one-way ANOVA with the least significant difference (LSD) was performed to compare the significant differences. The asterisks showed the *P* value significance level: *P < 0.05, **P < 0.01, ***P < 0.001, and ****P < 0.0001. Abbreviations used are as follows: BF for broad-leaved forest, CF for coniferous forest, CBF for mixed coniferous and broad-leaved forest, and MBF for mixed broad-leaved forest.

A total of 458 myxomycete OTUs were identified, including 84 OTUs within the order Stemonitales and 374 OTUs within the order Physarales (Fig. 3A). Taxonomic annotation via sequence similarity analysis resolved 111 OTUs (24.2% of total) to genus/species level, representing 16 genera and 40 morphospecies (Fig. 3A; Table S1). *Didymium* exhibited the highest OTU richness (49 OTUs), followed by *Physarum* (37 OTUs), *Diderma* (28 OTUs), *Stemonitis* (14 OTUs), *Macbrideola* (10 OTUs), and *Lamproderma* (9 OTUs). Rarefaction curves demonstrated near-complete sampling coverage across all communities, with extrapolated OTU recovery rates reaching 99.91–99.98% (Fig. 3B and C).

Myxomycete OTU compositions varied across forest types, with 297, 305, 263, and 297 OTUs obtained in MBF, CBF, CF, and MF, respectively. A core set of 150 OTUs was shared among forest types (Fig. 3D). Taxonomic analysis revealed that 16 annotated genera occurred in both MBF and BF, while *Craterium* was absent in CBF and *Lepidoderma* undetected in CF. Dominance patterns differed markedly: *Didymium* exhibited the highest relative abundance in MBF, CBF, and BF, while *Diderma* dominated in CF (Fig. 3A). One-way ANOVA revealed no statistically significant differences in OTU richness (*F* = 1.98, *P* = 0.12; Fig. 3E), Pielou's evenness index (*F* = 1.62, *P* = 0.19; Fig. 3F), and Shannon index (*F* = 0.86, *P* = 0.47; Fig. 3G). However, the LSD test revealed significantly higher OTU richness in BF (65.45 ± 22.13) compared to CF (50.05 ± 16.17, *P* < 0.05, Table 2).

**TABLE 2:** OTU richness, Pielou's evenness index, and Shannon index of myxomycete communities across forest types and seasons

		OTU richness	Pielou's evenness index	Shannon index	Goods coverage
Forest type	MBF	58.10 ± 22.77	0.48 ± 0.15	1.91 ± 0.59	99.95% ± 0.02%
	CBF	55.45 ± 19.65	0.53 ± 0.12	2.09 ± 0.51	99.95% ± 0.02%
	CF	50.05 ± 16.17	0.54 ± 0.14	2.10 ± 0.52	99.95% ± 0.01%
	BF	65.45 ± 22.13	0.46 ± 0.14	1.88 ± 0.58	99.94% ± 0.02%
Season	Spring	50.70 ± 14.54	0.41 ± 0.46	1.58 ± 0.60	99.95% ± 0.01%
	Summer	79.05 ± 16.76	0.54 ± 0.09	2.33 ± 0.38	99.93% ± 0.01%
	Autumn	36.00 ± 6.32	0.60 ± 0.11	2.16 ± 0.40	99.97% ± 0.01%
	Winter	63.30 ± 13.92	0.47 ± 0.12	1.92 ± 0.51	99.94% ± 0.02%

Seasonal dynamics significantly influenced the myxomycete community structure, with 237, 348, 185, and 288 OTUs obtained in spring, summer, autumn, and winter, respectively. A core set of 103 OTUs was shared among all seasons (Fig. 3E). Sixteen annotated genera all occurred in summer, while *Lepidoderma* and *Polyschisium* were absent in spring, *Craterium*, *Badhamia*, *Lepidoderma*, and *Mucilago* were absent in autumn, and *Lepidoderma* was absent in winter. *Didymium* consistently dominated seasonal assemblages with the highest relative abundance, followed by *Diderma* and *Physarum* (Fig. 3A). One-way ANOVA revealed significant seasonal differences in OTU richness (*F* = 36.97, *P* < 0.01; Fig. 3E), Pielou's evenness index (*F* = 9.98, *P* < 0.01; Fig. 3F), and Shannon index (*F* = 9.05, *P* < 0.01; Fig. 3G). LSD test further delineated these patterns: summer exhibited the highest OTU richness (79.05 ± 16.76) and Shannon index (2.327 ± 0.38), whereas autumn showed the highest Pielou's evenness index (0.60 ± 0.11; Table 2). Conversely, autumn exhibited the lowest OTU richness (36.00 ± 6.32), whereas the lowest Pielou's evenness index (0.41 ± 0.46) and Shannon index (1.578 ± 0.60) were recorded in spring (Table 2).

### β Diversity of myxomycete community

**Fig 4:**
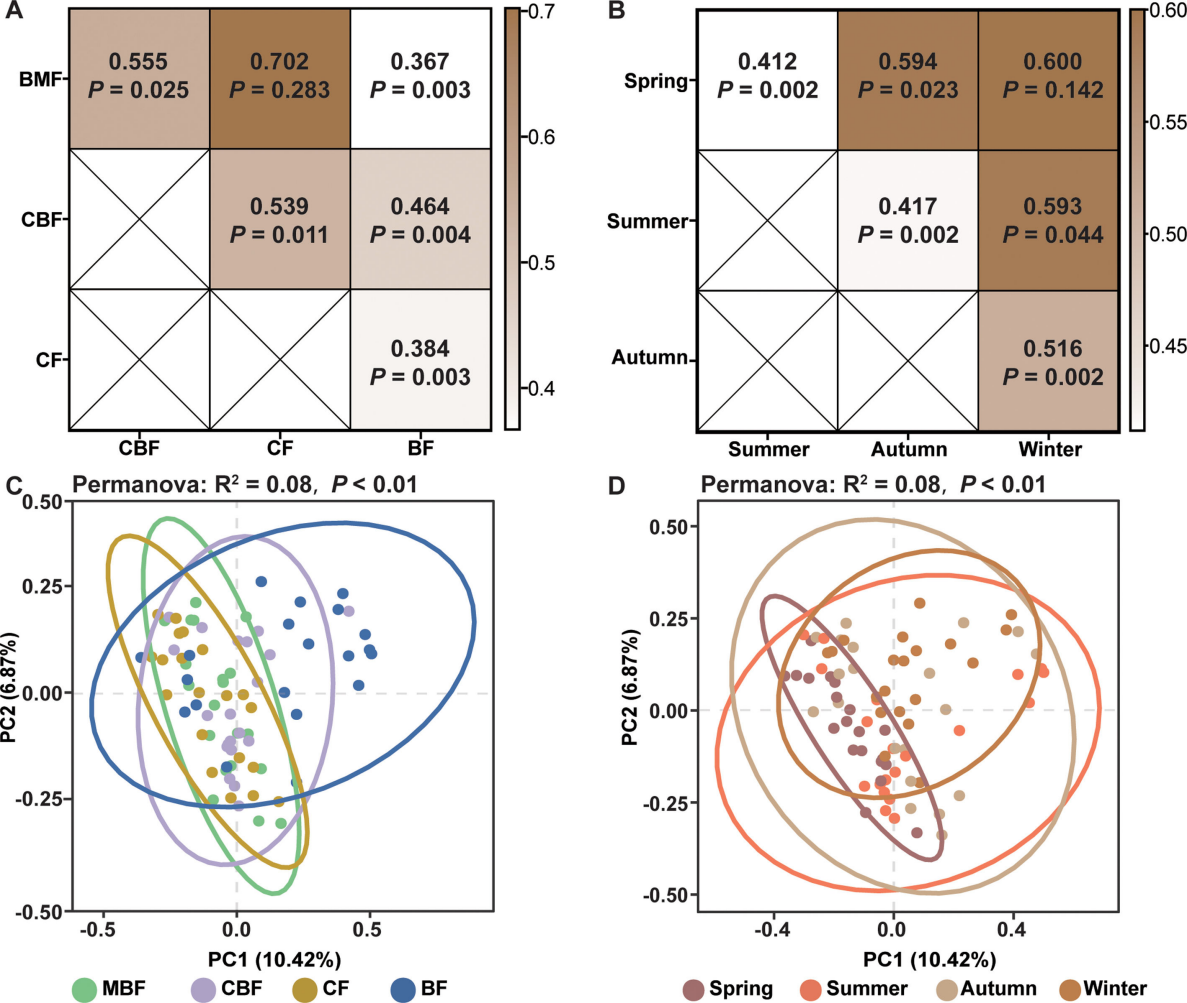
β Diversity of soil myxomycete communities. (**A and B**) Heatmap displays the Morisita-Horn similarity index (top value) and *P* values (bottom) of pairwise PERMANOVA for comparisons between forest types (**A**) and seasons (**B**), with color gradients reflecting the magnitude of similarity (darker hues = higher similarity) and *P* values indicating statistical significance of community dissimilarities. (**C and D**) Principal coordinate analyses ranking based on Bray-Curtis distance of the myxomycete communities across forest types (**C**) and seasons (**D**). Abbreviations used are as follows: BF for broad-leaved forest, CF for coniferous forest, CBF for mixed coniferous and broad-leaved forest, and MBF for mixed broad-leaved forest.

Comparisons based on the Morisita-Horn similarity index revealed low similarity in myxomycete community composition across four forest types (Fig. 4A). The highest similarity was observed between BMF and CF (MH = 0.702), whereas the lowest similarity occurred between BMF and BF (MH = 0.384). PCoA based on the Bray-Curtis distance revealed distinct community clustering among forest types, with particularly strong differentiation between CF, MBF, and BF (Fig. 4C). Samples from CF and MBF clustered more closely. In contrast, samples from BF and CBF showed great dispersion across the PCoA ordination. PERMANOVA confirmed significant differences among forest types (*F* = 2.10, R^2^ = 0.08, and *P* < 0.01). Pairwise PERMANOVA (Fig. 4A) comparisons corroborated both PCoA patterns and MH index results, showing a homogeneous myxomycete community between MBF and CF (*P* = 0.283) but significant differences among other forest types (*P* < 0.05).

Seasonal comparisons via the Morisita-Horn similarity index revealed generally low similarity among the four seasons (Fig. 4B). Spring and winter exhibited the strongest similarity (MH = 0.600), while spring and summer showed the weakest similarity (MH = 0.412). PCoA based on the Bray-Curtis distance showed that samples from spring tended to be more closely related to each other. Conversely, samples from summer, autumn, and winter demonstrated broader spatial distribution in the PCoA ordination (Fig. 4D). PERMANOVA indicated significant seasonal compositional variation (*F* = 2.13, R^2^ = 0.08, *P* < 0.01). Pairwise PERMANOVA (Fig. 4B) confirmed a homogeneous myxomycete community between spring and winter (*P* = 0.142) but significant differences between all other season pairs (*P* < 0.05).

### Myxomycete community assembly processes

**Fig 5:**
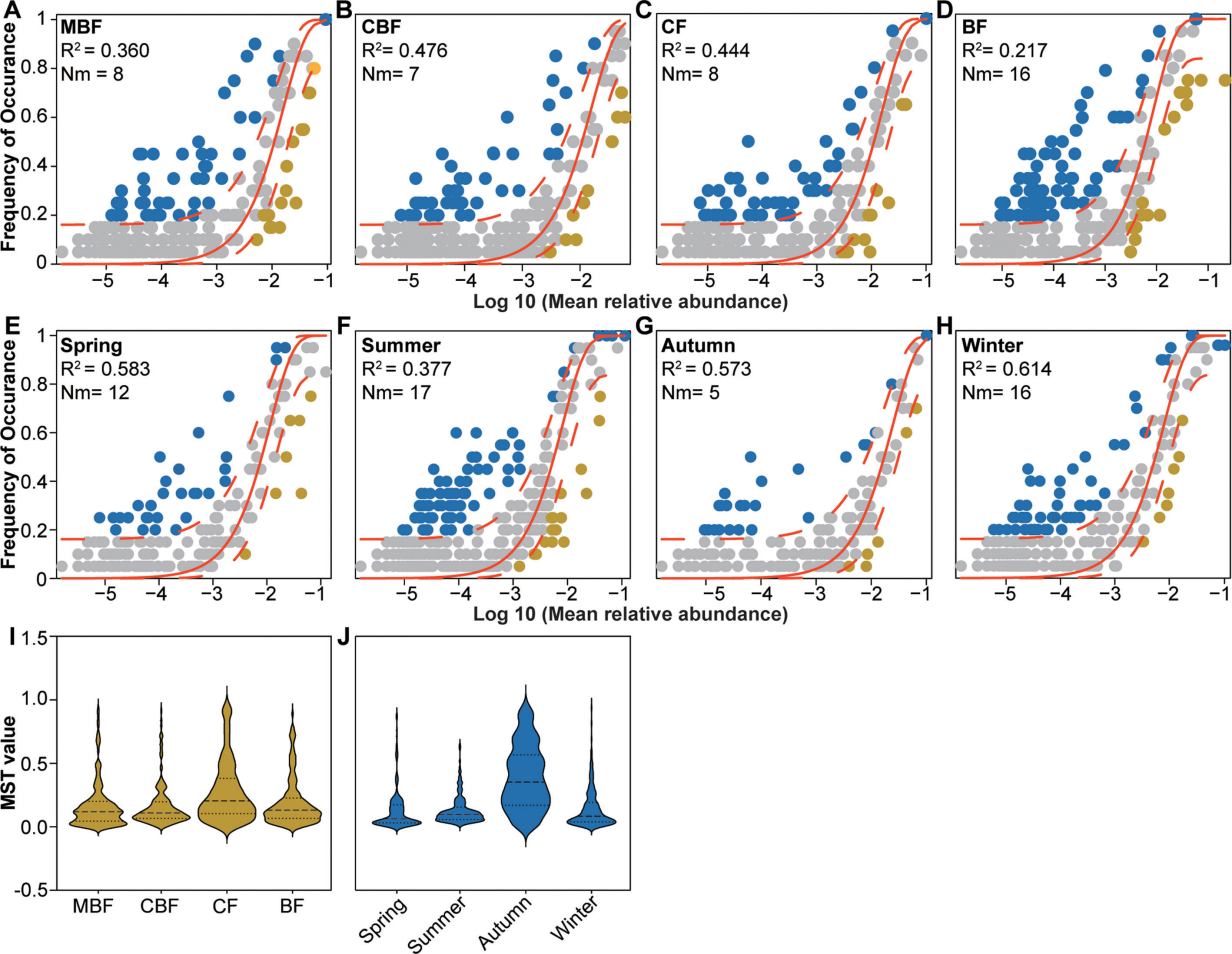
Sloan's neutral community models (A–H) and modified stochasticity ratio (**I and J**) for myxomycete community across forest types and seasons. In Sloan's neutral community models, R^2^ (coefficient of determination) reflects the model fit, Nm = meta-community (N) × migration rate (m), the solid red line represents the best fit of the model, and the red dashed line represents the 95% CI of the model's prediction. OTU distributions show the following: blue = observed frequencies exceeding model predicted, yellow = frequencies below predictions, and gray = frequencies within expected ranges. Abbreviations used are as follows: BF for broad-leaved forest, CF for coniferous forest, CBF for mixed coniferous and broad-leaved forest, and MBF for mixed broad-leaved forest.

The R^2^ of Sloan's neutral community model showed a trend of CBF (0.476) >CF (0.444) >MBF (0.360) >BF (0.217) (Fig. 5A through D), suggesting that deterministic processes predominantly governed myxomycete community assembly. Seasonal analysis showed a similar pattern, with R^2^ sequentially from winter (0.614) to spring (0.583), autumn (0.573), and summer (0.377) (Fig. 5E through H), indicating heightened deterministic processes selection during colder seasons. Notably, the elevated Nm values in BF and summer suggest low dispersal limitation and higher migration rates compared to other forest types and seasons.

Similarly, the modified stochasticity ratio confirmed that deterministic selection was the primary driver of community assembly across both forest types (Fig. 5I) and seasons (Fig. 5J). Average modified stochasticity ratio values were quantified as follows: (1) forest type: 0.17 ± 0.19 for MBF, 0.17 ± 0.17 for CBF, 0.28 ± 0.25 for CF, and 0.20 ± 0.19 for BF; and (2) season: 0.13 ± 0.18 for spring, 0.13 ± 0.11 for summer, 0.40 ± 0.26 for autumn, and 0.14 ± 0.16 for winter.

### Relationships between environmental factors and the myxomycete community

**Fig 6:**
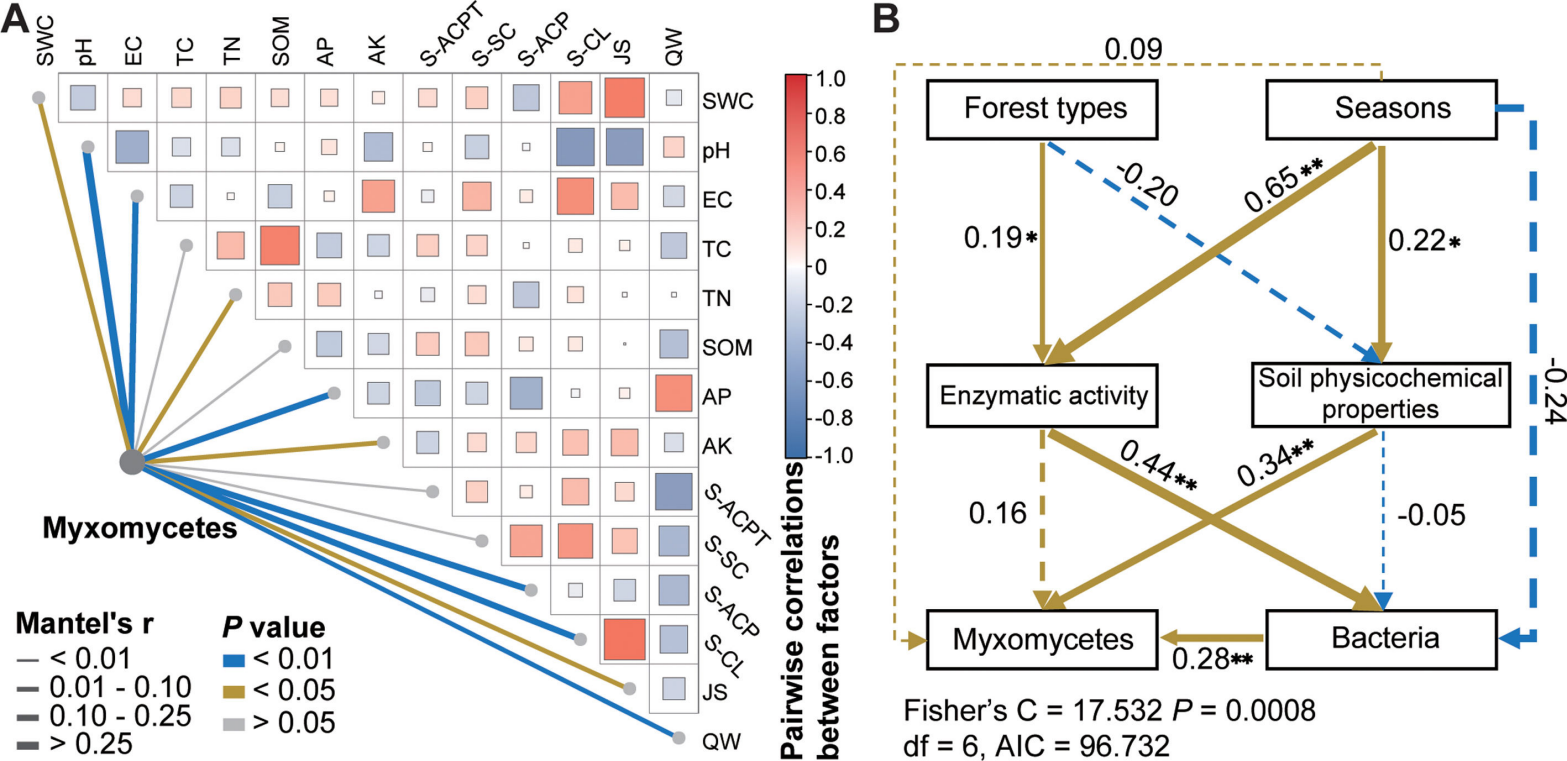
The interrelationships between soil myxomycete community, bacterial community, soil physicochemical properties, enzyme activity, and season. (**A**) Correlation analysis of soil myxomycete communities with soil physicochemical properties, enzyme activity, and seasons. (**B**) Structural equation modeling linking forest type, season, soil enzyme activity, and soil properties on soil myxomycete and bacterial communities. The asterisks show the *P* value significance level: *P < 0.05, **P < 0.01, ***P < 0.001, and ****P < 0.0001. Abbreviations used are as follows: SWC for soil water content, EC for soil electrical conductivity, TC for soil total carbon, TN for soil total nitrogen, SOM for soil organic matter SOM, AP for soil available phosphorus, AK for soil available potassium, S-ACPT for soil acid proteinase activities, S-SC for soil invertase activities, S-ACP for soil acid phosphatase activities, S-CL for soil cellulase activities, JS for quarterly average precipitation, and QW for quarterly average temperature.

Mental test based on Spearman correlation revealed significant associations between soil myxomycete community and multiple environmental variables: moderate correlations with SWC, TN, AK, and JS (*P* < 0.05) and stronger correlations with pH, EC, AP, S-ACP, and S-CL (*P* < 0.01) (Fig. 6A). SEM further delineated cascading ecological linkages: forest type (R^2^ = 0.19, *P* < 0.05) and season (R^2^ = 0.65, *P* < 0.01) significantly direct effects on soil enzyme activity, which subsequently mediated substantial shifts in bacterial community (R^2^ = 0.44, *P* < 0.01). Bacterial community further proves effects on myxomycete community (R^2^ = 0.28, *P* < 0.01). Additionally, seasonal dynamics directly modulated soil physicochemical properties (R^2^ = 0.22, *P* < 0.05), establishing a significantly secondary pathway influencing soil myxomycete community (R^2^ = 0.34, *P* < 0.01) (Fig. 6B).

### Potential interactions between myxomycetes and bacteria

**Fig 7:**
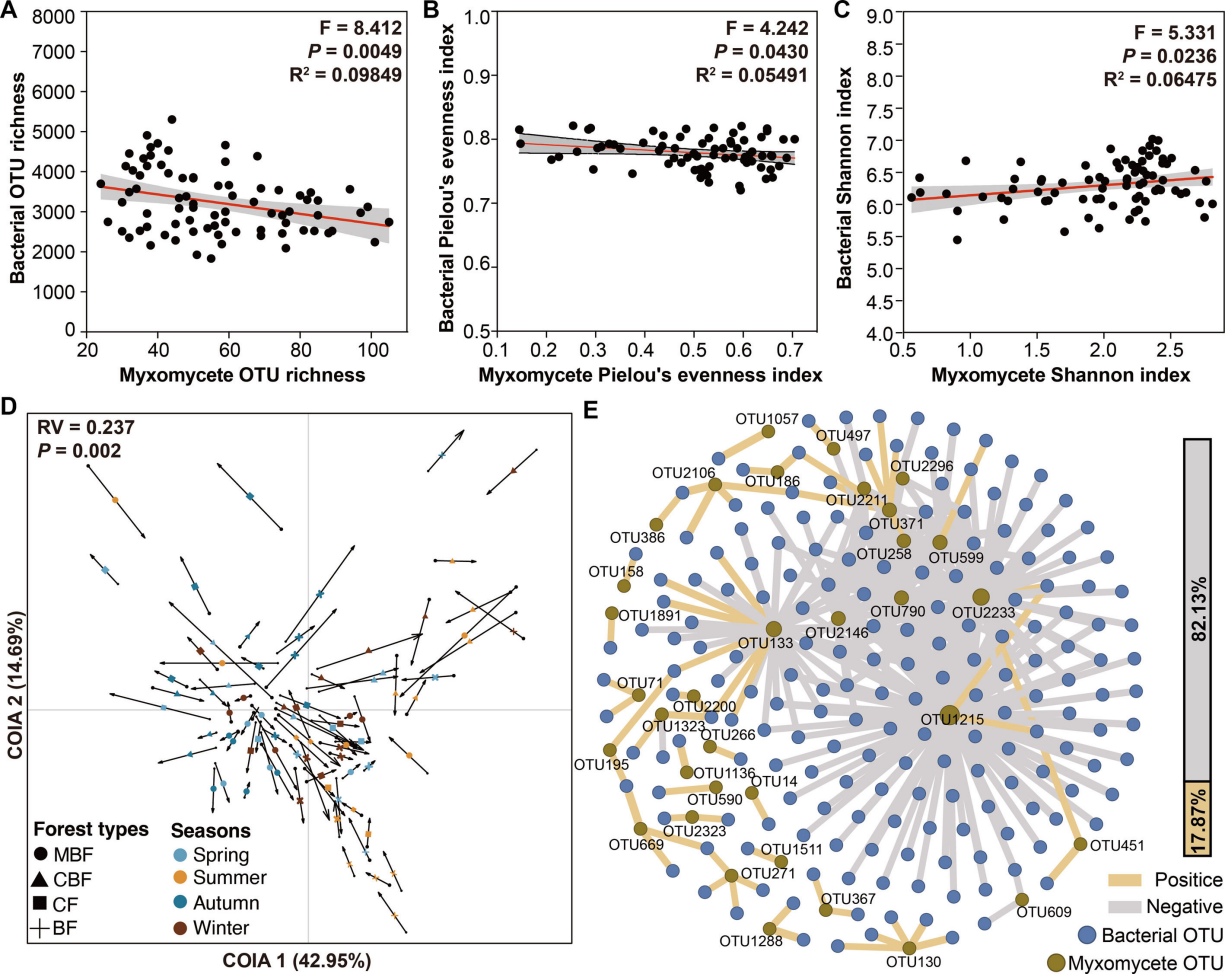
(A–C) Linear regression analysis of myxomycete and bacteria diversity indices: OTU richness (**A**), Pielou's evenness index (**B**), and Shannon index (**C**). (**D**) Co-inertia analysis of myxomycete and bacterial communities. RV represents the coefficient of correlation. The beginning of the arrow (black closed circles) is the position of each soil sample described by myxamoeba, and the end of the arrow is the position of each soil sample described by bacteria, with lengths reflecting inter-group divergence. (**E**) Co-occurrence network showing significant myxomycetes and bacteria associations (|Spearman's r| > 0.5, adjusted *P* < 0.05). Nodes are colored by taxa and sized by the number of connections (degree). Edges colored by interaction type (yellow: positive; grey: negative). Abbreviations used are as follows: BF for broad-leaved forest, CF for coniferous forest, CBF for mixed coniferous and broad-leaved forest, and MBF for mixed broad-leaved forest.

A strong negative correlation was observed between the myxomycete OTU richness and bacterial OTU richness (R^2^ = 0.09848, *P* < 0.01; Fig. 7A), as well as between myxomycete Pielou's evenness index and bacterial Pielou's evenness index (R^2^ = 0.05491, *P* < 0.05; Fig. 7B). Moreover, a significant positive correlation was observed between myxomycete Shannon index and bacterial Shannon index (R^2^ = 0.06475, *P* < 0.05; Fig. 7C). Co-inertia analysis revealed a significant synergistic covariation between myxomycete and bacterial communities (RV = 0.237, *P* < 0.01; Fig. 7D), with the first and second axes explaining 42.95% and 14.69% of the total projected inertia, respectively. The arrow lengths in the co-inertia analysis did not show a clear pattern of variation across forest types and seasons (Fig. S1), indicating that forest types and seasons were not determinants of the concordance between myxomycete and bacterial communities.

**Fig 8:**
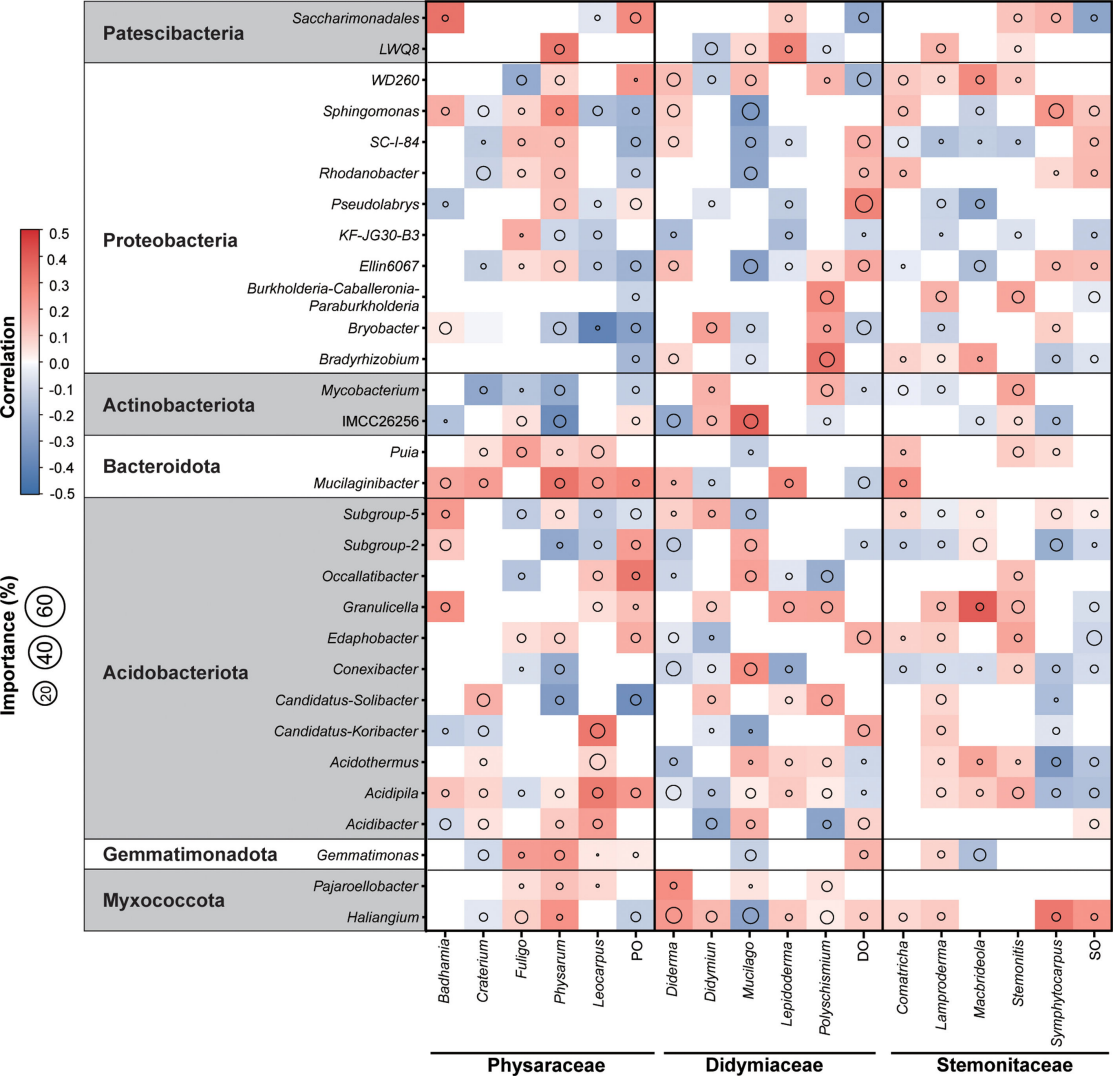
Importance of bacteria genus in predicting myxomycete genus. The circle color represents Spearman's correlation strength, and size reflects the variable importance by the random forest model (i.e., %IncMSE).

Random forest analysis (Fig. 8) indicates that many bacterial genera are the most important predictive factors for soil myxomycete. Within the Physaraceae, abundance patterns showed the strongest associations with *Mucilaginibacter* (Bacteroidota), *Candidatus-Koribacter*, and *Acidipila* (Acidobacteriota). *Saccharimonadales* (Patescibacteria) demonstrated predictive efficacy for *Badhamia*, while *Fuligo* exhibited the strongest correlation with *Puia. Physarum* abundance covaried with *LWQ8* (Patescibacteria), *Mycobacterium*, and *IMCC26256* (Actinobacteriota). A strong positive correlation emerged between *Candidatus-Koribacter* (Acidobacteriota) and *Leocarpus*. Didymiaceae abundance was mainly predicted by *Sphingomonas*, *Pseudolabrys,* and *Bradyrhizobium* (Proteobacteria), complemented by *IMCC26256* (Actinobacteriota) and *Haliangium* (Myxococcota). Notably, *IMCC26256* showed significant positive correlation with *Mucilago*, while *Haliangium* emerged as a key predictor for *Diderma*, *Didymium*, and *Mucilago*. Stemonitaceae abundance was primarily explained by Actinobacteriota and Acidobacteriota. *Granulicella* demonstrated superior predictive performance for *Macbrideola*, whereas *Sphingomonas* showed optimal predictive capacity for *Symphytocampus* abundance.

After Benjamini-Hochberg correction for multiple comparisons, 347 myxomycete-bacterial OTU pairs showed significant ecological associations (|r| > 0.5). Most of these interactions demonstrated negative correlations (82.13%, 285 pairs, r = −0.50 to −0.70), while only 17.87% (62 pairs, r = 0.50 to 0.60) showed positive relationships (Fig. 7E; Table S2). At the myxomycete OTU level, OTU1215 (*Didymium melanospermum*) exhibited the strongest interaction breadth, demonstrating correlations with 100 bacterial OTUs across five phyla: Proteobacteria (35, including two positive correlations), Acidobacteriota (23), Thermoleophilia (12), Bacteroidota (21), Firmicutes (5, including one positive correlation), and Desulfobacterota (1). The other myxomycete OTUs that have shown significant correlation with bacterial OTUs are: OTU2233 (56, belongs to Physaraceae), OTU133 (38, *Diderma spumarioides*), OTU599 (24, belongs to *Physarum*), OTU790 (20, belongs to Didymiaceae), OTU371 (20, belongs to Stemonitidaceae), OTU2146 (18, *Badhamia capsulifera*), and OTU258 (13, *Physarum melleum*). For bacterial taxa, at the phylum level, Bacteroidia demonstrated the highest associations (118 pairs), followed by Alphaproteobacteria (69), Acidobacteriae (31), and Thermoleophilia (28). At the family level, Muribaculaceae emerged as the most interactive bacterial taxa (79 pairs).

## DISCUSSION

### Richness and taxonomic composition of northern subtropical managed forests

High-throughput sequencing of 80 soil samples, combined with stringent quality filtering protocols, a total of 458 myxomycete OTUs, were obtained in this study using primers S31R/S3b. This primer system demonstrates exceptional specificity for the dark-spore clade (mainly Physarales and Stemonitales) (46, 47), allowing for the identification of robust constituent genera and species. Previous validation studies have demonstrated its superior performance in amplifying diverse dark-spored clades across multiple soil ecosystems (2, 7, 9, 10, 30). Notably, the OTU richness obtained in this study surpasses values reported for soil myxomycetes in the German Alps (30), northwestern Russia (2), and Henan Province, China (9), but it is comparable to the numbers of myxomycete OTUs obtained in subtropical China (7) and the Chinese bright coniferous forest (8). This indicates significant differences in soil myxomycete composition across broader scales.

At the genus level, taxonomic annotations revealed that the genera *Didymium*, *Diderma,* and *Physarum* of the family Physaraceae dominate soils in the northern subtropical managed forest (Fig. 3). This dominance differed from previous studies where the genera *Lamproderma*, *Diderma*, and *Meriderma* were dominant (2, 11, 30). At the species level, this study successfully annotated 61 OTUs to 40 morphospecies (Table S1), surpassing previous reports by a substantial margin in soil myxomycete diversity studies (2, 8, 9, 11, 30). This advancement may be predominantly ascribed to recent progress in myxomycete taxonomy and the refinement of group-specific PCR primers, particularly those targeting the family Physaraceae (48, 49). Enhanced molecular reference databases now enable more precise matching of environmental OTUs to described species, overcoming historical limitations in sequence annotation. Additionally, the pronounced climatic and vegetative heterogeneity across sampling regions likely contributed to the observed spatial divergence in OTU richness and community structure (16). It should be noted that several nivicolous myxomycetes, such as nivicolous *Lamproderma* species (*L. pulchellum*, *L. pseudomaculatum*, *L. ovoideum*, and *L. echinosporum*) and *Polyschismium chailletii*, were detected in this study (Table S1). The ecological guild of nivicolous myxomycetes includes about 100 species, almost all of which belong to the dark-spored myxomycete clade (50). This limited nivicolous myxomycetes diversity in Zijin Mountain National Forest Park may be because the geographical area is affected by the subtropical humid monsoon climate (21, 22), without thick long-lasting snow cover, and does not create stable conditions for the development of rich nivicolous myxomycete communities (30, 51).

### Spatiotemporal patterns of soil myxomycete communities

Seasonal dynamics are widely recognized as key drivers of microbial community structure and diversity in soil ecosystems (52, 53). However, the extent to which these temporal patterns govern soil myxomycetes remains poorly understood. Current research has primarily focused on periods of active myxomycete proliferation, with limited research across full annual cycles. Previous reports indicate temporal fluctuations in myxomycete communities, exemplified by OTU disappearance in June samples and subsequent reappearance in September samples, alongside a 13.9% community composition variation attributed to sampling month (2). In this study, our finding reveals distinct seasonal patterns in myxomycete communities, with winter and spring communities demonstrating notable similarity in both composition (represented by Morisita-Horn similarity index and PCoA analysis; Fig. 4B and D). Moreover, summer soil exhibited peak OTU richness and Shannon index (Fig. 3F and H), while autumn soil exhibited peak Pielou's evenness index (Fig. 3G) and reduced richness and Shannon index without significant diversity loss relative to summer. This pattern potentially reflects seasonal transitions between plasmodial and fruiting body stages during unfavorable spring/winter conditions. Contrasting with these results, a study in Baotianman Nature Reserve reported no significant seasonal variations in myxomycete communities (9). This discrepancy may stem from methodological differences, as their study excluded winter sampling while our four-season analysis captured broader temporal dynamics. Additionally, divergent outcomes across studies likely arise from site-specific variables, including various factors such as vegetation composition, microclimate conditions, and edaphic properties. Such context-dependent variations underscore the necessity for expanded longitudinal monitoring across biogeographic regions to disentangle universal seasonal patterns from localized ecological effects in myxomycete community ecology.

However, this seasonal difference was not reflected in the process of community assembly. Fruiting body-based studies of myxomycete diversity indicate that, while most species exhibit broad geographic distributions, certain taxa demonstrate distinct microhabitat preferences, offering empirical support for the interplay of deterministic and stochastic processes in community assembly (16, 54). Early research on soil myxomycete communities reported predominantly stochastic assembly patterns across and within vegetation types (10). Subsequent investigations, however, have demonstrated that deterministic processes also play an important role (7). Our study employed Sloan’s neutral community model and modified the stochasticity ratio to disentangle assembly processes across forest types and seasons. Sloan's neutral community model indicates neutral processes as the non-negligible drive of soil myxomycete assemblage (R^2^ consistently exceeded 0.2, reaching 0.6 in some cases; Fig. A-D, F-I). However, the incomplete explanatory power of Sloan's neutral community model (R^2^ < 1.0) implies the concurrent operation of other assembly processes and mechanisms, such as environmental filtering and biotic interactions. The modified stochasticity ratio further suggested deterministic dominance, with modified stochasticity ratio values persistently below 0.5 across all soil samples. This may arise from spatially heterogeneous abiotic conditions (e.g., microclimate, edaphic factors) and biotic landscapes at sampling sites, which collectively drive divergent community structures and modulate process hierarchies (8).

### Effects of enzyme activity on soil myxomycete communities

Soil biological factors, such as interactions among bacteria, fungi, and other microorganisms, as well as the secretion of their products, can make soil abiotic factors unpredictable. Soil enzyme activity can reflect the activity of soil microorganisms, and it is one of the important biological activity indicators for evaluating soil quality, as well as an important biological factor influencing soil microorganisms. This study explored for the first time the influence of enzyme activity on the structure of myxomycete communities. Mantel tests showed that S-CL and S-ACP activities were highly correlated with the soil myxomycete community (*P* < 0.01; Fig. 6A), implying an important role of myxomycetes in soil *P* and C cycles. SEM (Fig. 6B) showed that soil enzyme activity directly affected the effect of myxomycete community compared to other factors, but soil enzyme activity significantly influenced bacterial community (R^2^ = 0.44, *P* < 0.01), and bacterial community significantly influenced myxomycete community (R^2^ = 0.28, *P* < 0.01). Therefore, the specific relationship and interaction mechanisms between enzyme activity and myxomycete communities still need further exploration.

### Relationship between soil myxomycetes and bacteria

Myxomycetes are typically considered bacterivores (13, 14), and the potential predation and prey-predator interactions between myxomycetes and bacteria can be inferred from previous studies (7, 10, 15). In this study, linear regression models demonstrated a significant negative correlation between myxomycete OTU richness and bacterial OTU richness (Fig. 7A), as well as between their respective Pielou's evenness indices (Fig. 7B), contrasting with a significant positive correlation between their Shannon indices (Fig. 7C). This further suggests that the relationship between myxomycetes and bacteria may be more complex than a simple predator-prey relationship (15) and that there may be a trade-off between species diversity of myxomycetes and bacteria (Fig. 7A). The overall diversity change shows opposite trends (Fig. 7C), and there is an opposing relationship between the two in terms of community distribution uniformity (Fig. 7B). This indicates that there are multiple interactions between myxomycetes and bacteria, which may be dominated by resource competition. Co-inertia analysis revealed statistically significant covariation between myxomycete and bacterial communities (RV = 0.237, *P* < 0.01; Fig. 7D), demonstrating measurable ecological linkage between these trophic groups. Notably, this covariation pattern exhibited cross-ecosystem stability, showing no discernible modulation by forest type and seasonal variation (Fig. S1). This finding implies that potential environment-independent biological interactions may operate through mechanisms less susceptible to extrinsic environmental filters. However, the precise nature of these cross-kingdom relationships, whether driven by trophic interactions, metabolic dependencies, or alternative ecological mechanisms, remains unresolved. Further experimental studies are required to dissect the directionality and molecular underpinnings of myxomycete-bacterial interactions, particularly their reciprocal influences under varying ecological contexts.

Previous studies have established that myxomycete communities exhibit positive ecological correlations with bacterial phyla such as Proteobacteria, Actinobacteriota, and Bacillales (10), suggesting a potential active recruitment of specific bacteria taxa from environment reservoirs (15). Our study reveals distinct family-level association patterns that refine previous observations of myxomycete-bacteria relationships (10). Members of Physaraceae showed preferential linkages with Bacteroidota (a group enriched in polysaccharide degraders) and Actinobacteriota (noted for secondary metabolite production), members of Didymiaceae association with Proteobacteria, Actinobacteriota, and Myxococcota (predatory bacteria sharing similar microhabitats), and members of Stemonitaceae dynamic appeared predominantly influenced by Actinobacteriota and Proteobacteria (Fig. 8). These taxon-specific association patterns reinforce the concept of myxomycetes as a trophically specialized group within Amoebozoa (10), functioning primarily as microbial consumers in soil ecosystems (14) with bacterial prey availability serving as a key population driver.

The overwhelming prevalence of negative correlations (82.13% of significant OTU pairs; Fig. 7E; Table S2) provided critical quantitative support for the predator-prey dynamics hypothesis (14). Notably, myxomycete OTU1215 (*D. melanospermum*) demonstrated exceptional interaction breadth through 100 bacterial associations, yet 98% were negative, a pattern mirroring the “optimal foraging” strategy where generalist predators maintain diverse prey contacts without stable mutualisms (55, 56). This contrasts with the limited positive correlations (17.87%; Fig. 7E; Table S2), which clustered disproportionately in Proteobacteria. Bacterial taxa showed phylum-level predominance in Bacteroidia (118 pairs) and Alphaproteobacteria (69), with Muribaculaceae emerging as the most interactive family (79 pairs). The observed network complexity prompts a fundamental question: do these correlations primarily stem from active selection behaviors in slime molds coupled with differential survival capabilities among bacteria, or could they be artifacts of undetected abiotic variables in the microenvironment? This ambiguity highlights crucial knowledge gaps in understanding the evolutionary drivers shaping these cross-domain associations.

## Data Availability

The raw data generated in this study were deposited in the NCBI Sequence Read Archive under accession numbers PRJNA1241702 (Myxomycetes) and PRJNA1241720 (Bacteria).

## SUPPLEMENTAL MATERIAL

Figure S1 aem.00479-25-s0001.docx Statistical results of the arrow length in the co-inertia analysis (CoIA) between myxomycete communities and bacterial communities of four forest types and four seasons.

Supplemental legends aem.00479-25-s0002.docx Legends for Fig. S1 and Tables S1 and S2.

Table S1 aem.00479-25-s0003.docx Species annotated according to similarity greater than 98%.

Table S2 aem.00479-25-s0004.xlsx Co-occurrence network showing significant myxomycetes and bacteria associations.

ASM does not own the copyrights to Supplemental Material that may be linked to, or accessed through, an article. The authors have granted ASM a non-exclusive, world-wide license to publish the Supplemental Material files. Please contact the corresponding author directly for reuse.
