# Supplementary material for: Spatiotemporal patterns of soil myxomycetes in subtropical managed forests and their potential interactions with bacteria
Source: Appl Environ Microbiol. 2025 May 13;91(6):e00479-25. doi: 10.1128/aem.00479-25 (PMC12175498; doi:10.1128/aem.00479-25)
Supplement: Figure S1 — Statistical results of the arrow length in the co-inertia analysis (CoIA) between myxomycete communities and bacterial communities of four forest types and four seasons. [file aem.00479-25-s0001.docx]

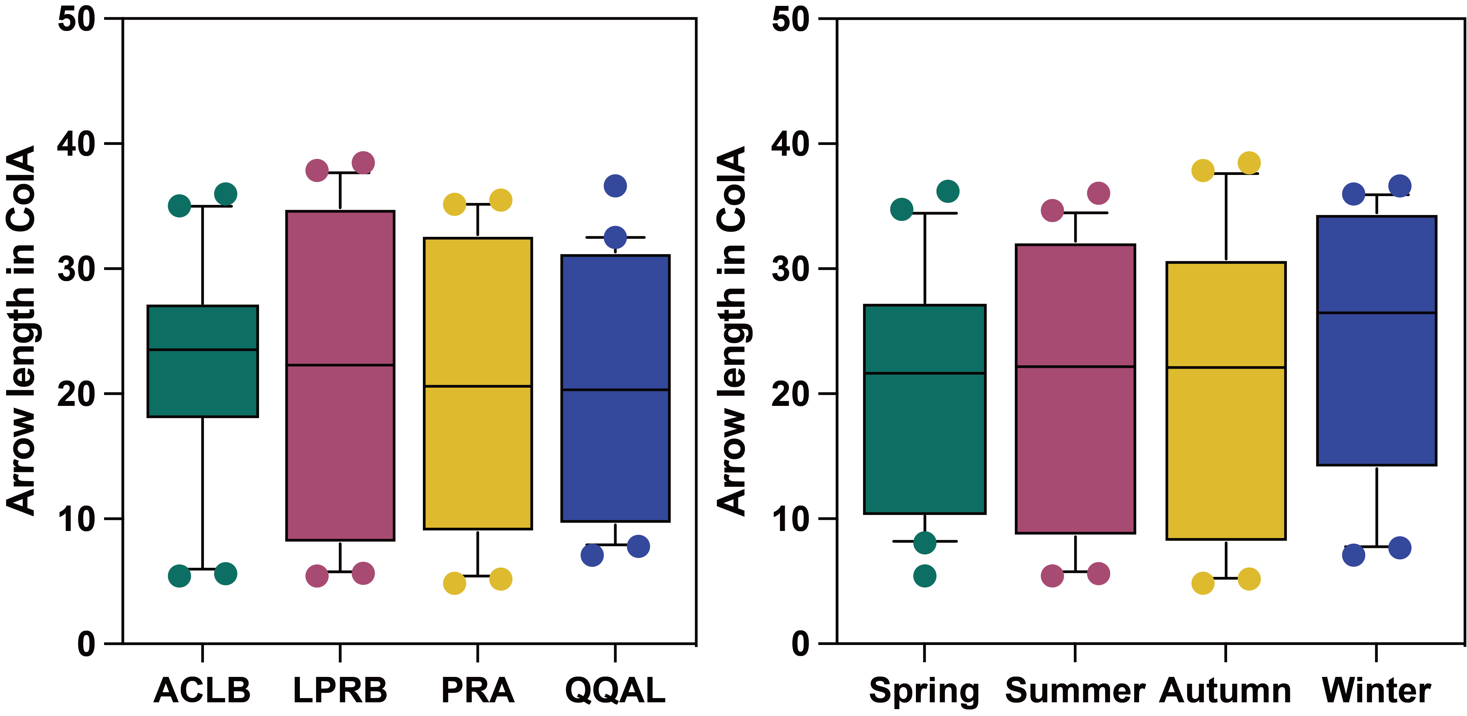


**FIG S1** The statistical results of the arrow length in the Co-inertia analysis (CoIA) between myxomycete communities and bacterial communities of four forest types (A) and four seasons (B).
