# Supplementary material for: Spatiotemporal patterns of soil myxomycetes in subtropical managed forests and their potential interactions with bacteria
Source: Appl Environ Microbiol. 2025 May 13;91(6):e00479-25. doi: 10.1128/aem.00479-25 (PMC12175498; doi:10.1128/aem.00479-25)
Supplement: Supplemental legends — Legends for Fig. S1 and Tables S1 and S2. [file aem.00479-25-s0002.docx]

# Supplemental material legend

**FIG S1** The statistical results of the arrow length in the Co-inertia analysis (CoIA) between myxomycete communities and bacterial communities of four forest types (A) and four seasons (B).

**TABLE S1** Species annotated according to similarity greater than 98%.

**TABLE S2** Co-occurrence network showing significant myxomycetes and bacteria associations (|Spearman’s r| >0.5, adjusted *P* < 0.05).
