# Supplementary material for: Spatiotemporal patterns of soil myxomycetes in subtropical managed forests and their potential interactions with bacteria
Source: Appl Environ Microbiol. 2025 May 13;91(6):e00479-25. doi: 10.1128/aem.00479-25 (PMC12175498; doi:10.1128/aem.00479-25)
Supplement: Table S1 — Species annotated according to similarity greater than 98%. [file aem.00479-25-s0003.docx]

**TABLE S1** Species annotated according to similarity greater than 98%.

| Myxomycete OTU ID | OTU taxonomy | Similarity | NCBI number |
| --- | --- | --- | --- |
| 36 | *Symphytocarpus impexus* | 98.70% | AY230188 |
| 560 | *Symphytocarpus amaurochaetoides* | 98.70% | MH930799 |
| 1549 | *Stemonitis pallida* | 99.70% | MK041083 |
| 1310 | *Stemonitis pallida* | 98.85% | MT846406 |
| 283 | *Stemonitis pallida* | 99.09% | MK041083 |
| 257 | *Stemonitis pallida* | 98.72% | MT846406 |
| 19 | *Stemonitis herbatica* | 99.71% | MK041080 |
| 666 | *Stemonitis foliicola* | 100.00% | MK041077 |
| 1307 | *Lamproderma scintillans* | 98.76% | MZ241467 |
| 1394 | *Lamproderma pulchellum* | 98.73% | MZ005930 |
| 696 | *Lamproderma pseudomaculatum* | 98.70% | MN913624 |
| 459 | *Lamproderma ovoideum* | 100.00% | OP621244 |
| 102 | *Lamproderma ovoideum* | 100.00% | OP621244 |
| 873 | *Lamproderma echinosporum* | 100.00% | OP679846 |
| 1820 | *Lamproderma columbinum* | 100.00% | HQ687203 |
| 630 | *Comatricha pulchella* | 100.00% | MH930536 |
| 24 | *Comatricha pulchella* | 98.82% | MH930536 |
| 2203 | *Comatricha nigra* | 100.00% | MT846406 |
| 579 | *Comatricha nigra* | 100.00% | MT846404 |
| 1804 | *Physarum sessile* | 99.16% | MW693018 |
| 1097 | *Physarum rigidum* | 100.00% | HE614604 |
| 2342 | *Physarum melleum* | 98.91% | KF743870 |
| 2265 | *Physarum melleum* | 98.57% | MH930742 |
| 1879 | *Physarum melleum* | 100.00% | MH930742 |
| 1182 | *Physarum melleum* | 98.75% | MN722595 |
| 786 | *Physarum melleum* | 98.57% | MH930742 |
| 783 | *Physarum melleum* | 99.07% | MN722595 |
| 767 | *Physarum melleum* | 98.57% | MH930742 |
| 544 | *Physarum melleum* | 100.00% | MG647879 |
| 430 | *Physarum melleum* | 98.19% | MG647882 |
| 258 | *Physarum melleum* | 100.00% | MG647908 |
| 397 | *Physarum leucophaeum* | 98.97% | MH930740 |
| 2106 | *Physarum globuliferum* | 98.90% | MF352475 |
| 414 | *Physarum clavisporum* | 98.73% | MF352472 |
| 1719 | *Physarum auriscalpium* | 98.66% | ON870404 |
| 591 | *Physarum album* | 98.48% | MG647917 |
| 669 | *Leocarpus fragilis* | 100.00% | MN334125 |
| 1961 | *Fuligo septica* | 99.72% | MH348906 |
| 1707 | *Craterium leucocephalum* | 99.40% | OP621205 |
| 2146 | *Badhamia capsulifera* | 100.00% | OP621196 |
| 735 | *Polyschismium peyerimhoffii* | 100.00% | MN334015 |
| 1368 | *Polyschismium chailletii* | 98.98% | KY123415 |
| 663 | *Polyschismium chailletii* | 98.70% | OP621279 |
| 1571 | *Didymium squamulosum* | 100.00% | MH348904 |
| 1057 | *Didymium squamulosum* | 98.91% | OP621235 |
| 553 | *Didymium squamulosum* | 98.86% | OP621236 |
| 123 | *Didymium squamulosum* | 99.72% | MH348904 |
| 1427 | *Didymium radiaticolumellum* | 98.96% | ON059429 |
| 1557 | *Didymium quitense* | 100.00% | MG662516 |
| 1959 | *Didymium nigripes* | 98.78% | MH930807 |
| 888 | *Didymium nigripes* | 99.08% | MH930807 |
| 325 | *Didymium minus* | 98.10% | KU577272 |
| 285 | *Didymium minus* | 98.50% | KF743865 |
| 1215 | *Didymium melanospermum* | 100.00% | MH930568 |
| 13 | *Didymium flexuosum* | 100.00% | KM977857 |
| 133 | *Diderma spumarioides* | 98.98% | MH714791 |
| 95 | *Diderma saundersii* | 100.00% | MG696636 |
| 1034 | *Diderma effusum* | 99.07% | MZ604988 |
| 75 | *Diderma deplanatum* | 98.47% | KM977851 |
| 531 | *Diderma alpinum* | 100.00% | MN595609 |
| 478 | *Diderma alpinum* | 100.00% | MN595609 |
